# Loss of FGFR3 Delays Acute Myeloid Leukemogenesis by Programming Weakly Pathogenic CD117-Positive Leukemia Stem-Like Cells

**DOI:** 10.3389/fphar.2020.632809

**Published:** 2021-01-29

**Authors:** Chen Guo, Qiuju Ran, Chun Sun, Tingting Zhou, Xi Yang, Jizhou Zhang, Shifeng Pang, Yechen Xiao

**Affiliations:** ^1^ Department of Biotechnology, Guangdong Medical University, Dongguan, China; ^2^ Department of Biochemistry and Molecular Biology, College of Basic Medical Science, Jilin University, Changchun, China

**Keywords:** leukemia (acute myeloid), stem-like cancer cells, fibroblast growth factor receptor 3, FGFR1, erg, CCL3 chemokine

## Abstract

Chemotherapeutic patients with leukemia often relapse and produce drug resistance due to the existence of leukemia stem cells (LSCs). Fibroblast growth factor receptor 3 (FGFR3) signaling mediates the drug resistance of LSCs in chronic myeloid leukemia (CML). However, the function of FGFR3 in acute myeloid leukemia (AML) is less understood. Here, we identified that the loss of FGFR3 reprograms MLL-AF9 (MA)-driven murine AML cells into weakly pathogenic CD117-positive leukemia stem-like cells by activating the FGFR1-ERG signaling pathway. FGFR3 deletion significantly inhibits AML cells engraftment *in vivo* and extends the survival time of leukemic mice. FGFR3 deletion sharply decreased the expression of chemokines and the prolonged survival time in mice receiving FGFR3-deficient MA cells could be neutralized by overexpression of CCL3. Here we firstly found that FGFR3 had a novel regulatory mechanism for the stemness of LSCs in AML, and provided a promising anti-leukemia approach by interrupting FGFR3.

## Introduction

Acute myeloid leukemia (AML) is a malignant disease with a heterogeneous molecular panel of mutations participating in the blockade of differentiation and the uncontrolled clonal proliferation of myeloid hematopoietic stem and progenitor cells (Ferrara and Schiffer, 2013). Chemotherapeutic patients with AML often relapse and produce drug resistance due to the existence of leukemia stem cells (LSCs) (Fernandez et al., 2019). LSCs in AML represent a minor fraction at the apex of a cellular hierarchy which initiates and maintains the disease, exhibiting properties of self-renewal, cell cycle quiescence, and drug resistance (Siveen et al., 2017). It is urgent to find the regulatory mechanism of LSCs so that AML could be cured by eliminating LSCs.

Fibroblast growth factors (FGFs) play important roles in regulating cell proliferation, differentiation, and survival during embryo development and the adult stage by the FGF receptor (FGFR1-4)-mediated signaling pathway (Wesche et al., 2011). Previous studies showed that FGF signaling is very essential for promoting hematopoietic stem cell (HSC) expansion *in vitro* and *in vivo* (de Haan et al., 2003). FGF1 was also used for the *in vitro* culture of LSCs (Peng et al., 2010). Nevertheless, how FGFs affect biological functions remains unclear. FGFR3, an important member of the FGFR family, is highly expressed in HSCs (Dvorak and Hampl, 2005), and increased in LSCs (CD34^+^BCR-ABL^+^) from chronic myeloid leukemia (CML) (Dvorak et al., 2003). FGF2 induces imatinib resistance in patients’ CD34^+^ CML cells via reactivation of FGFR3-RAS-MAPK signaling (Traer et al., 2014). A previous study showed that the FGFR3 fusion protein (TEL-FGFR3) induced T-lymphoma and subsequently progressed to AML (Maeda et al., 2005). Up to date, there have been few reports on the relationship between FGFR3 and AML as well as AML-LSCs.

In this study, an MLL-AF9 (MA)-transduced AML cell model was constructed to study the functions of FGFR3 signaling in AML. We found that FGFR3 deletion reprogrammed MA-AML cells into CD117^+^ leukemia stem-like cells by activating the FGFR1-ERG-CD117 signaling pathway. We also found that FGFR3-deficient leukemia cells were weakly pathogenic due to the downregulation of chemokines genes. Collectively, we firstly identified a novel regulatory mechanism of FGFR3 in AML and provided evidence supporting its clinical significance in AML.

## Methods

### Mice

*FGFR3* ^*−/−*^ mice on a C57BL/6 background were constructed via CRISPR/Cas9-mediated genome engineering and provided by Cyagen Biosciences Inc (Suzhou, China). The C57BL/6 mice were purchased from Changsheng Inc. (Benxi, China). All mice used in this study were housed in the animal facility at the College of Basic Medical Sciences of Jilin University and handled according to the guidelines of Animal Care and Use Committee of Jilin University.

### Genotyping

Genomic DNA was extracted from mice tails as described previously (Xiao et al., 2011), and the FGFR3 gene fragment was amplified by PCR. The PCR product was detected by 1.5% agarose electrophoresis, and confirmed for DNA sequencing (Sangon, Shanghai, China). To identify the FGFR3 knockout (KO) mouse model, three primers were designed as follows: FGFR3-F (5′-CTG​TGT​CCG​CCT​AGT​GGA​GG AGC-3′), FGFR3-R (5′-GAA​CGG​AGG​AGG​CGG​GGG​TT-3′), and FGFR3-Wt/He-R (5′-AGTGCCAAG AAG​GGT​TTC​AGA​CAA​C-3′), respectively.

### Expression Plasmid Construction, Virus Packing, and Infection

The genes used in this study, including ERG (mouse), CCL3 (mouse), and constitutively active FGFR1 (CA-FGFR1, human) (Petiot et al., 2002) were synthesized and cloned into retrovirus vector MSCV-GFP by ZooNBio Biotechnology (Nanjing, China).

For retrovirus construction, the MSCV-GFP or MSCV-MLL-AF9 plasmid and Gag-pol plasmid were both transfected into 293T cells by liposome (Jinchuan, Changchun, China), and the supernatant was collected at 36 h and 72 h, respectively. The virus was concentrated by a centrifugal filter (Millipore, USA). CD117^+^ cells from murine BM were selected by magnetic beads according to the method of the EasySep mouse CD117-positive screening kit (STEMCELL Technologies Inc., Canada), then they were infected with the retrovirus containing different genes at 32°C centrifugation for 4 h, and the GFP^+^ cells were sorted by flow cytometry. For lentivirus construction, lentivirus containing a short hairpin RNA (shRNA) plasmid against mouse ERG (LV-U6 > mERG [shRNA]-mPGK > mCherry/T2A/Puro), was provided by Cyagen Biosciences Inc (Guangzhou, China). The target sequence of mouse ERG (CGA​CAT​TCT​TCT​CTC​ACA​T) was simply adapted from the human ERG gene as described previously (Sun et al., 2008; Tsuzuki et al., 2011).

### Cell Culture

For primary BM CD117^+^ cells, the cells were cultured in DMEM medium containing 15% bovine fetus serum, 100 ng/ml of SCF, 10 ng/ml of IL3, and 10 ng/ml of IL6. For primary MLL-AF9-transduced cells, the cells were cultured in DMEM medium containing 10% bovine fetus serum, 20 ng/ml of SCF, 10 ng/ml of IL3, and 10 ng/ml of IL6.

### Mouse AML Cell Transplantation

For the primary transplantation assays, MA-transduced cells were injected by the tail vein into lethally irradiated (8.5 Gy) 8 to 10-week-old C57BL/6 recipient mice with 2 × 10^5^ donor cells per mouse plus a radioprotective dose of 5 × 10^5^ whole BM cells. For the secondary transplantation assays, 2 × 10^4^ leukemic cells isolated from the bone marrow or spleen of primary recipient mice were further transplanted into lethally irradiated C57BL/6 secondary recipient mice. The survival of recipient mice was monitored over time and analyzed by Kaplan-Meier survival graphing.

### Flow Cytometry and Antibodies

BM, SP, and PB were collected from the different types or different treatments of the mice. MNCs were isolated from these tissues after red blood cell lysis. Cells were suspended in fluorescence-activated cell sorting buffer (PBS supplemented with 2% FBS) at a concentration of 1 × 10^7^ cells/ml and aliquoted into flow cytometry tubes (100–200 μL per tube) for antibody staining. The analysis was performed with flow cytometry (BD LSRFortessa ^TM^ or BD Accuri ^TM^ C6) as described previously (Xiao et al., 2011). Antibodies used in this study were purchased from eBioscience, including Gr1-FITC (RB6-8C5), B220-FITC (RA3-6B2), Ter119-FITC (TER-119), CD11b-FITC (M1/70), CD3e-FITC (145-2C11), Gr1-PE (RB6-8C5), CD3e-PE (145-2C11), Ter119-PE (TER-119), ISO Control-PE (Ebr2a), CD11b-PE (M1/70), CD117-PE (2B8), B220-PE (RA3-6B2), CD11b-APC (M1/70), CD117-APC (ACK2), Gr1-APC (RB6-8C5), CD11b-PECy5.5 (M1/70), CD4-PECy5.5 (RM4-5), B220-PECy5.5 (RA3-6B2), CD4-PECy5 (GK1.5), CD11b-PECy5 (M1/70), B220-PECy5 (RA3-6B2), and CD8a-PECy5 (53–6.7). The secondary antibody (Goat pAb to Rb IgG/PE, ab72465) was purchased from Abcam.

### RNA Sequencing Analysis

For RNA sequencing (RNA-seq) analysis of the leukemia cells, 2 × 10^6^ MA-WT cells or MA-KO cells were stored in TRIzol solution and delivered to Sangon Biotech (Shanghai, China) for RNA-seq analysis using the Illumina Hiseq2000 Genome Analyzer instrument (Illumina, San Diego, CA). The analysis was performed as described previously (Yao et al., 2019).

### RNA Isolation, cDNA Synthesis, and Quantitative Real-Time PCR

Total RNA from different types of cells was extracted using TRIzol reagent (Thermo) according to the manufacturer’s instruction, and cDNA was prepared using a reverse transcription (RT) kit (TransGen Biotech, Beijing, China). Quantitative real-time PCR analysis was performed by a SYBR Green kit (TransGen Biotech) using a MX3000 P system. The expression level of each gene was quantified and normalized to the *β*-actin expression using the following primer pairs (all mouse):

CXCL10 forward: 5′-GTC​ATT​TTC​TGC​CTC​ATC​CT-3'; CXCL10 reverse: 5′-GCC​CTT​TTA​GAC​CTT​TTT​TG-3'; CCL2 forward: 5′-GAT​CAG​AAC​CTA​CAA​CTT​TAT​T-3'; CCL2 reverse: 5′-TCA​CTG​TCA​CAC​TGG​TCA​CTC​C-3'; CCL3 forward: 5′-GCC​CTT​GCT​GTT​CTT​CTC​TGT​A-3'; CCL3 reverse: 5′-CGA​TGA​ATT​GGC​GTG​GAA​TCT​T-3'; CCL4 forward: 5′-TCT​CTC​TCC​TCT​TGC​TCG​TG-3'; CCL4 reverse: 5′-TGC​TGG​TCT​CAT​AGT​AAT​CC-3'; CCL5 forward: 5′-CTG​CTT​TGC​CTA​CCT​CTC​CCT​C-3'; CCL5 reverse: 5′-TCT​TGA​ACC​CAC​TTC​TTC​TCT​G-3'; CCR2 forward: 5′-GAA​TCT​TTT​TCA​TTA​TCC​TCC​T-3'; CCR2 reverse: 5′-GAC​TAC​ACT​TGT​TAT​CAC​CCC​A-3'; CCR5 forward: 5′-GTA​GGA​ATG​AGA​AGA​AGA​GGC​A-3'; CCR5 reverse: 5′-TCG​GAA​CTG​ACC​CTT​GAA​AAT​CCA​T-3'; MESIS1 forward: 5′-ATT​ATG​GGG​GTA​TGG​ATG​GAG​T-3'; MESIS1 reverse: 5′-TGC​TAA​GAG​AGG​GAA​GAG​GGG​G-3'; FGFBP3 forward: 5′-AAG​CTC​CCA​GCC​GGA​AAA​GAA​G-3'; FGFBP3 reverse: 5′-GTT​GAC​AAA​GAA​GTT​GCA​AAG​G-3'; STAT5a forward: 5′-TGG​GGG​AAG​ATG​GGT​TTT​TGC-3'; STAT5a reverse: 5′-CGT​TCT​CCG​TGT​CCT​GTG​TGA-3'; STAT5b forward: 5′-TGA​ACA​AGC​AAC​AGG​CCC​ACG​A-3'; STAT5b reverse: 5′-AAA​AGG​CAT​CAG​ATT​CCA​AAA​C-3'; HOXA9 forward: 5′-CCC​TGA​CTG​ACT​ATG​CTT​GTG​G-3'; HOXA9 reverse: 5′-ACC​TGC​CTT​TCG​GTG​AGG​TTG​A-3'; HOXA10 forward: 5′-CCC​TTC​GCC​AAA​TTA​TCC​CAC​A-3'; HOXA10 reverse: 5′-CAC​CAA​CAC​CAG​CCT​CGC​CTC​T-3'; HOXA11 forward: 5′-CGT​GGT​CCC​TGC​TCC​TCT​AAC​A-3'; HOXA11 reverse: 5′-GGG​CTC​AAT​GGC​GTA​CTC​TCT​G-3'; FGFR1 forward: 5′-AGA​CGG​ACA​ACA​CCA​AAC​CAA​A-3'; FGFR1 reverse: 5′-TCC​CAT​ACT​CAT​TCT​CCA​CGA​T-3'; FGF10 forward: 5′-TGT​TGC​TCT​TTT​TGG​TGT​CTT-3'; FGF10 reverse: 5′-CTC​CGA​TTT​CCA​CTG​ATG​TTA-3'; PBX3 forward: 5′-CTC​CGA​GAA​CAG​AGT​AGA​A-3'; PBX3 re-verse: 5′-GCT​GAG​GTG​TGA​GTA​AAA​A-3'; ERG forward: 5′-AGT​GTG​AAC​AAA​GGT​GGG​AAG​A-3'; ERG reverse: 5′-AGT​CAG​ATG​TGG​AAG​GGG​AGT​C-3'; c-Kit forward: 5′-ACA​AAA​GCA​ACA​TCA​GAT​AT-3'; c-Kit reverse: 5′-TGA​GAC​AGG​AGT​GGT​ACA​CC-3'; Cyclin D1 forward: 5′-TCT​CCT​GCT​ACC​GCA​CAA​CGC-3'; Cyclin D1 reverse: 5′-TCC​ACT​TCC​CCC​TCC​TCC​TCA-3'; *β*-actin forward: 5′-GGA​GAT​TAC​TGC​CCT​GGC​TCC​TA-3'; *β*-actin reverse: 5′-GAC​TCA​TCG​TAC​TCC​TGC​TTG​CTG-3'.

### ChIP–PCR Assay

The plasmid MSCV-puro-ERG or MSCV-puro was transformed into HEK293 T cells, and the cells were cultured for 48 h. The subsequent Chromatin Immunoprecipitation (ChIP) assay was performed according to previously described methods (Jiao et al., 2017). The cells were crosslinked with 0.1% formaldehyde. Chromatin was broken down into 200–500 bp fragments using an ultrasonicator (Xinzhi, Ningbo, China). The HA antibody was added into the lysate and incubated overnight at 4°C, then ChIP–grade Protein A Dynabeads were added into each IP tube and incubated for 2 h. DNA was purified with the general method and eluted DNA was subjected to quantitative PCR (qPCR) to measure the enrichment according to the previous method. Primers against CD117 promoter region used for ChIP-PCR were designed as follows:

CD117-P1 forward: 5′-TTA​CAC​TTT​TAA​GCA​TTC​TG-3'; reverse: 5′-TGT​GTT​CTT​TCT​CTC​CAC​TG-3'. CD117-P2 forward: 5′-ATC​TTC​TCT​ACT​TCA​TAA​CG3'; reverse: 5′- TCT​ACC​TAG​GGA​CAT​TTC​AC-3'; CD117-P3 forward: 5′-AGT​AAA​ACA​CTC​ATA​CAC​AT-3'; reverse: 5′-TTT​TAA​TCT​CAC​CCT​CAA​CA-3'. CD117-P4 forward: 5′-AGC​CAG​CCG​CCT​GGA​CTG​AA-3'; reverse: 5′-CCT​AGT​GCC​CGG​GGC​TCT​CG-3'.

### Luciferase Reporter Assay

Using genomic DNA from mouse BM as a template, a 2000 bp DNA fragment, CD117p containing the CD117 promoter region was amplified by the PCR method as described previously (Li et al., 2017) and cloned into a pGL3-Basic plasmid, then three plasmids, including control plasmid pRL-TK, recombinant plasmid pGL3-CD117p or pGL3-Basic as well as MSCV-puro-ERG or MSCV-puro, were transfected into HEK293T cells, and the cells were cultured for 48 h. A luciferase reporter assay was performed as described previously (Jiang et al., 2019). The activity of firefly luciferase and Renilla luciferase was assayed using a Trans Detect Double-Luciferase Reporter Assay Kit (TransGen) in a multifunctional chemiluminometer. The relative luciferase activity was normalized by the ratio of firefly luciferase to Renilla luciferase for each transfected well. Each experiment was performed in quadruplicate and repeated three times.

### Colony-Forming/Replating Assays

A total of 1,500 different types of MA cells were seeded in the methylcellulose (MethoCult™ M3434, StemCell Technologies) and colonies were counted on day 7 of culture using an inverted microscope (Olympus). For the replating assay, the colonies were washed twice with PBS and centrifuged at 1,000 rpm for 5 min. The cells were resuspended with DEME medium and then 1,500 live cells were used for the colony-forming assay according to the previous description.

### Analysis of Chemokine Production in BM Supernatant by ELISA

BM cells from the femur and tibia of one mouse leg were washed with 1 ml of PBS, then the cells were removed by centrifugation and the BM supernatant was collected. For CCL3 and CCL4 detection, the standard working solution was diluted by gradient for the preparation of the standard curve (Elabscience ELISA kit). All experimental procedures were performed according to the kit instructions. When the reaction was terminated, the absorbance at a wavelength of 450 nm was immediately measured using a microplate reader.

### Western Blot and Antibodies

*In vitro* cultured cells or murine tissue samples were harvested and lysed with NP-40 (Thermo). Proteins from the lysate were fractionated by electrophoresis through 12% polyacrylamide gels and transferred onto PVDF membranes (Thermo) using Tris-Glycine Transfer buffer. The membrane was incubated overnight to block nonspecific binding with 5% nonfat milk powder, and incubated with the relative primary antibody (1:1,000) for 1 h, then washed and incubated with a 1:10,000 dilution of secondary HRP-conjugated antibody. Immunoreactive bands were visualized using an ECL kit (Thermo Scientific). For Western blotting and ChIP assays, the following antibodies were used: p-ERK (CST; #4370S; 1:1,000), ERK (Bioworld; BS1112; 1:1,000), AKT (CST; #9272S; 1:1,000), p-AKT (CST; 4060S; 1:1,000), FGFR1 (CST; 9740S; 1:1,000), p-FGFR1 (Affinity; AF3157; 1:1,000), ACTIN (CMCTAG; AT0001; 1:1,000), Goat Anti-Rabbit IgG (Bioworld; BS13278; 1:10,000), FGFR3 (NOVUS Biologicals; JM110–33; 1:1,000), ERG (abcam; ab92513; 1:1,000), HA Tag (Life Science; AB10004; 1:1,000).

### Statistical Analysis

Normally distributed experimental data were performed using the *Student*’*s t* test for single comparison and one-way or two-way ANOVA for multiple comparisons from GraphPad Prism Version 6 (GraphPad Software, La Jolla, CA, United States). The data were demonstrated using means ± SD and *p* < *0.05* was considered as statistically significant. *p < 0.05, **p < 0.01, ***p < 0.001, and ****p < 0.0001.

## Results

### Loss of FGFR3 in MA-Driven AML Cells Promotes the Generation of Weakly Pathogenic CD117-Positive Leukemia Stem-like Cells

**FIGURE 1:**
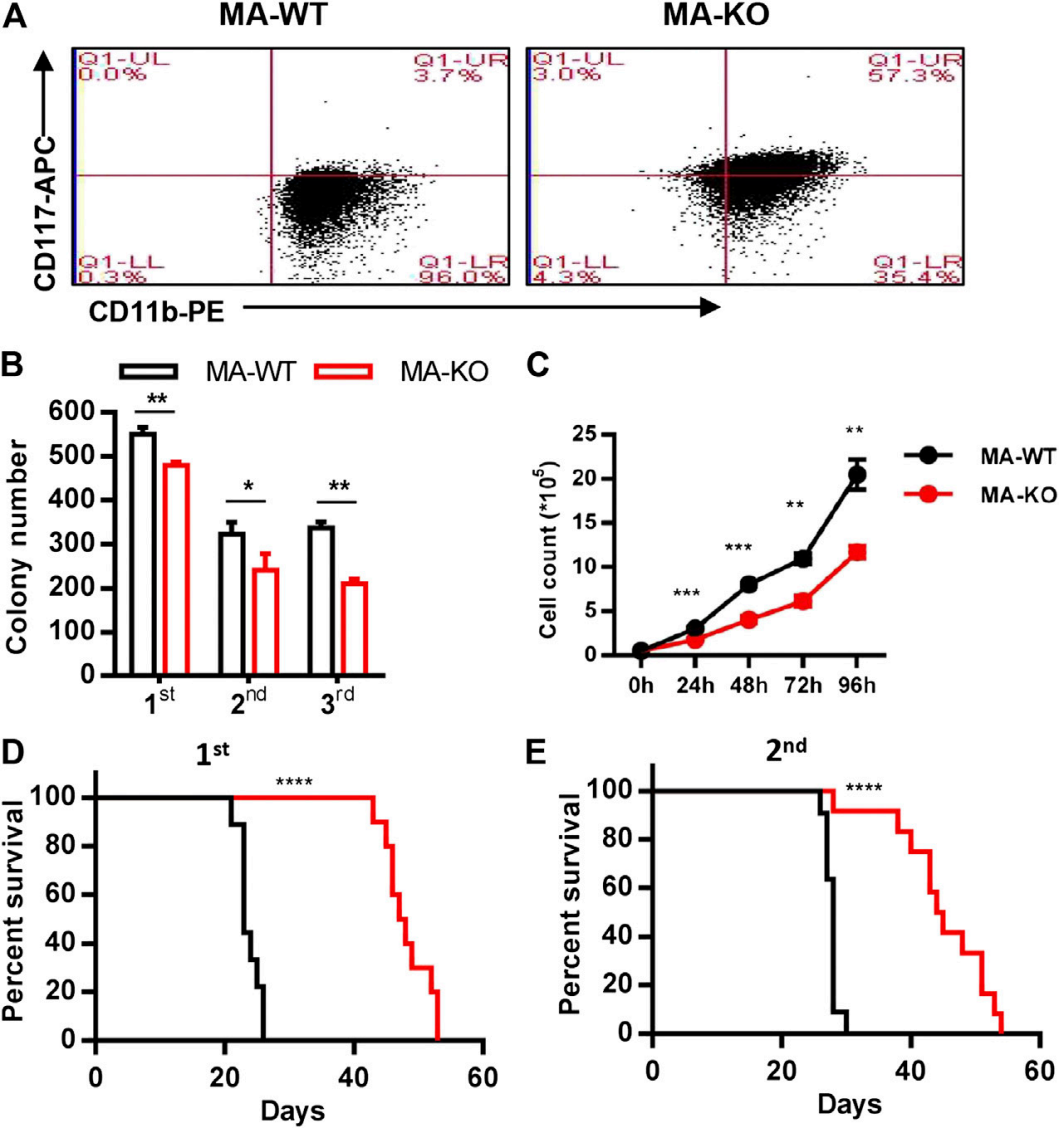
Loss of FGFR3 promotes the generation of weakly pathogenic CD117^+^ MLL-AF9-driven leukemia cells. **(A)** Representative flow cytometric analysis of MA-WT and MA-KO cells with CD117 and CD11b staining **(B)** *In vitro* colony-forming assay of MA-WT and MA-KO cells (n = 3). **(C)** Growth curve at indicated time from MA-WT and MA-KO cells (n = 5). **(D,E)** Effects of FGFR3 deletion in MLL-AF9-mediated *in vivo* leukemogenesis. Kaplan-Meier curves are shown for two groups of transplanted mice including MA-WT (n = 9), and MA-KO (n = 10) in a primary BMT assay **(D)**, and for two groups of transplanted mice including MA-WT (n = 11), and MA-KO (n = 12) in the secondary BMT assay **(E)**.*p < 0.05, **p < 0.01, ***p < 0.001, ****p < 0.0001.

To study whether FGFR3 is a potential target gene for AML therapy, MA-transduced pre-leukemia cells were constructed with CD117^+^ BM cells from *FGFR3* ^−/−^ and wild-type (WT) mice via infection of retrovirus (Supplementary Figures S1A,B). Leukemia cells were acquired from the BM or SP of leukemic mice which were transplanted with pre-leukemia cells according to the strategy (Supplementary Figure S1C). Firstly, we surprisingly found that CD117-positive leukemia stem-like cells (GFP^+^CD117^+^CD11b^+/low^) have a 20-fold higher percentage in MA-induced FGFR3-knockout leukemia cells (MA-KO) than in MA-induced WT leukemia cells (MA-WT) (Figure 1A). The colony forming assays of three continuous generations showed that MA-KO cells had lower growth rates than that of MA-WT cells (Figure 1B), which was also consistent with the *in vitro* cell growth curve at the indicated time (Figure 1C). Significantly delayed leukemia development and reduced leukemia-related mortality were observed in mice receiving MA-KO cells upon primary transplantation (Figure 1D) and secondary transplantation (Figure 1E) compared with the mice receiving MA-WT cells. These results showed that FGFR3 deletion in MA-driven AML cells inhibits cell proliferation and promotes the generation of weakly pathogenic CD117-positive leukemia stem-like cells.

### FGFR3 Deletion Does Not Affect the Homing Ability of MA-Leukemia Cells but Significantly Inhibits *In Vivo* Engraftment

**FIGURE 2:**
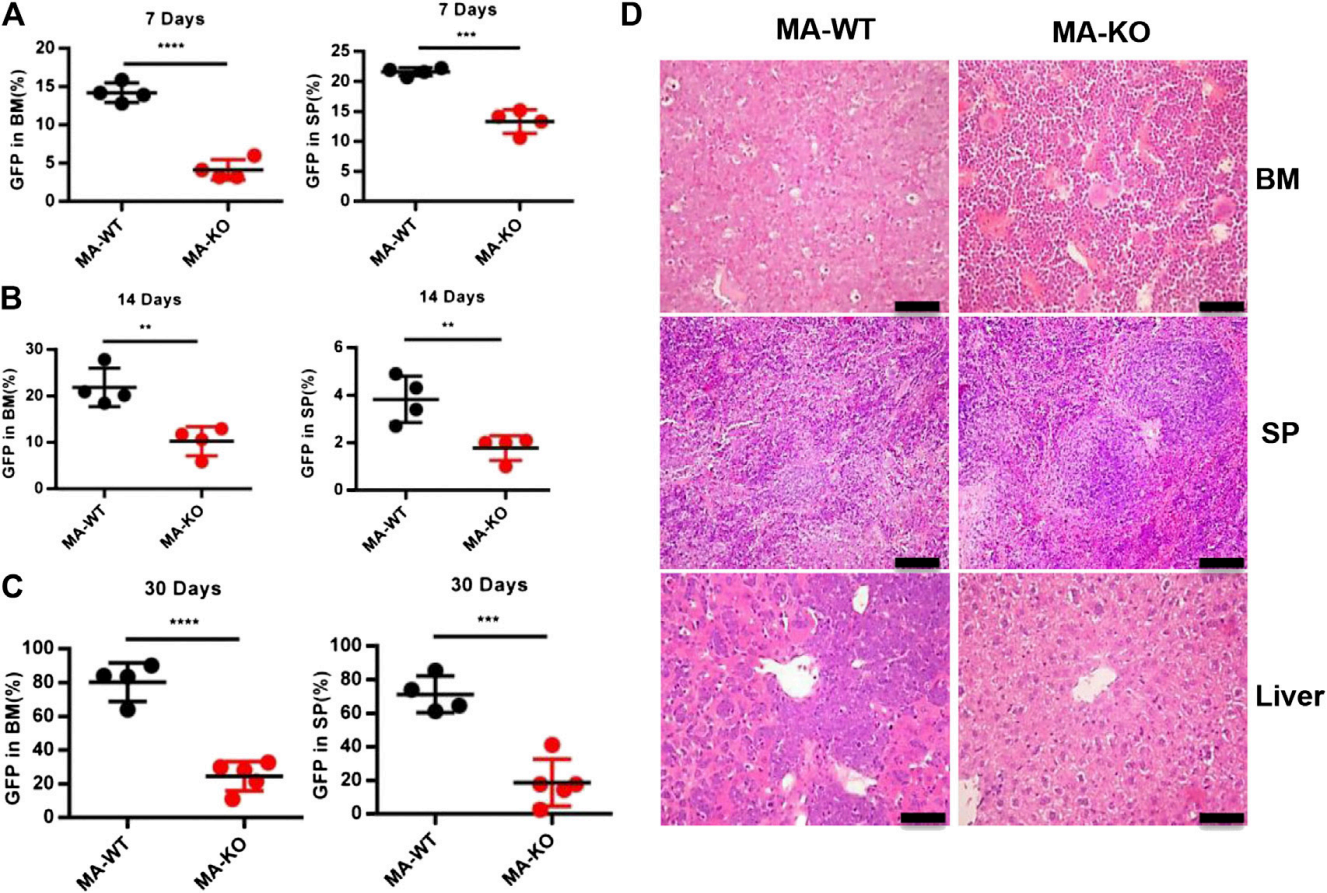
FGFR3 deletion significantly inhibits *in vivo* engraftment of leukemic mice. **(A–C)** The engraftment ability of GFP^+^ MA-WT and MA-KO cells from BM and SP at day 7 **(A)**, day 14 **(B)**, and day 30 **(C)** after transplantation. **(D)** Representative image of hematoxylin and eosin-stained sections at day 30 after transplantation of MA-WT and MA-KO cells. **p < 0.01, ***p < 0.001, ****p < 0.0001.

In order to define which stage affects mice survival, the homing ability of leukemia cells was analyzed at 24 h after transplantation, and the results showed that there was no significant difference in BM, SP, and PB in leukemic mice transplanted with MA-WT or MA-KO cells (Supplementary Figures S2A–C). However, the *in vivo* engraftment ability of MA-KO cells was remarkably lower than that of MA-WT cells at day 7, day 14, and day 30 (Figures 2A–C). Hematoxylin and eosin staining results confirmed that there were less infiltrative leukemia cells in BM, SP, and the liver of MA-KO transplanted mice than MA-WT transplanted mice at day 30 after transplantation (Figure 2D). These observations indicated that FGFR3 deletion attenuates the *in vivo* engraftment ability of MA-leukemia cells.

### FGFR3 Deletion Reprograms Leukemia Cells Into CD117-Positive Leukemia Stem-like Cell by Activating the FGFR1-AKT-ERG Signaling Pathway

**FIGURE 3:**
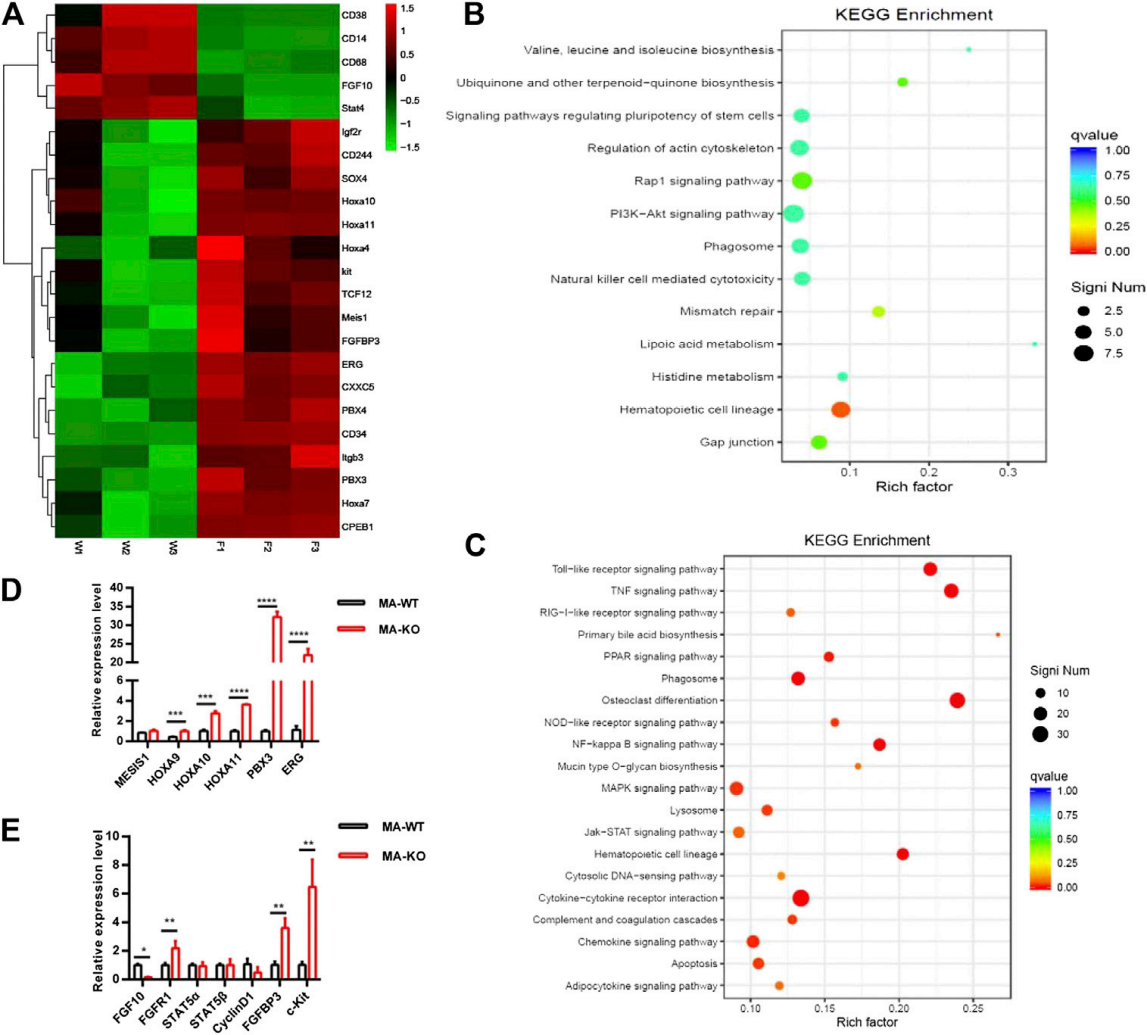
RNA-seq and differential gene analysis of MA-WT and MA-KO cells. **(A)** Heat maps showing gene expression kinetics from RNA-seq results of MA-WT samples (W1, W2, and W3) and MA-KO samples (F1, F2, and F3). **(B,C)** KEGG enrichment of pathway in MA-KO cells compared to MA-WT cells. **(D,E)** qRT-PCR assay of differential genes from **(A)** (n = 4). *p < 0.05, **p < 0.01, ***p < 0.001, ****p < 0.0001.

**FIGURE 4:**
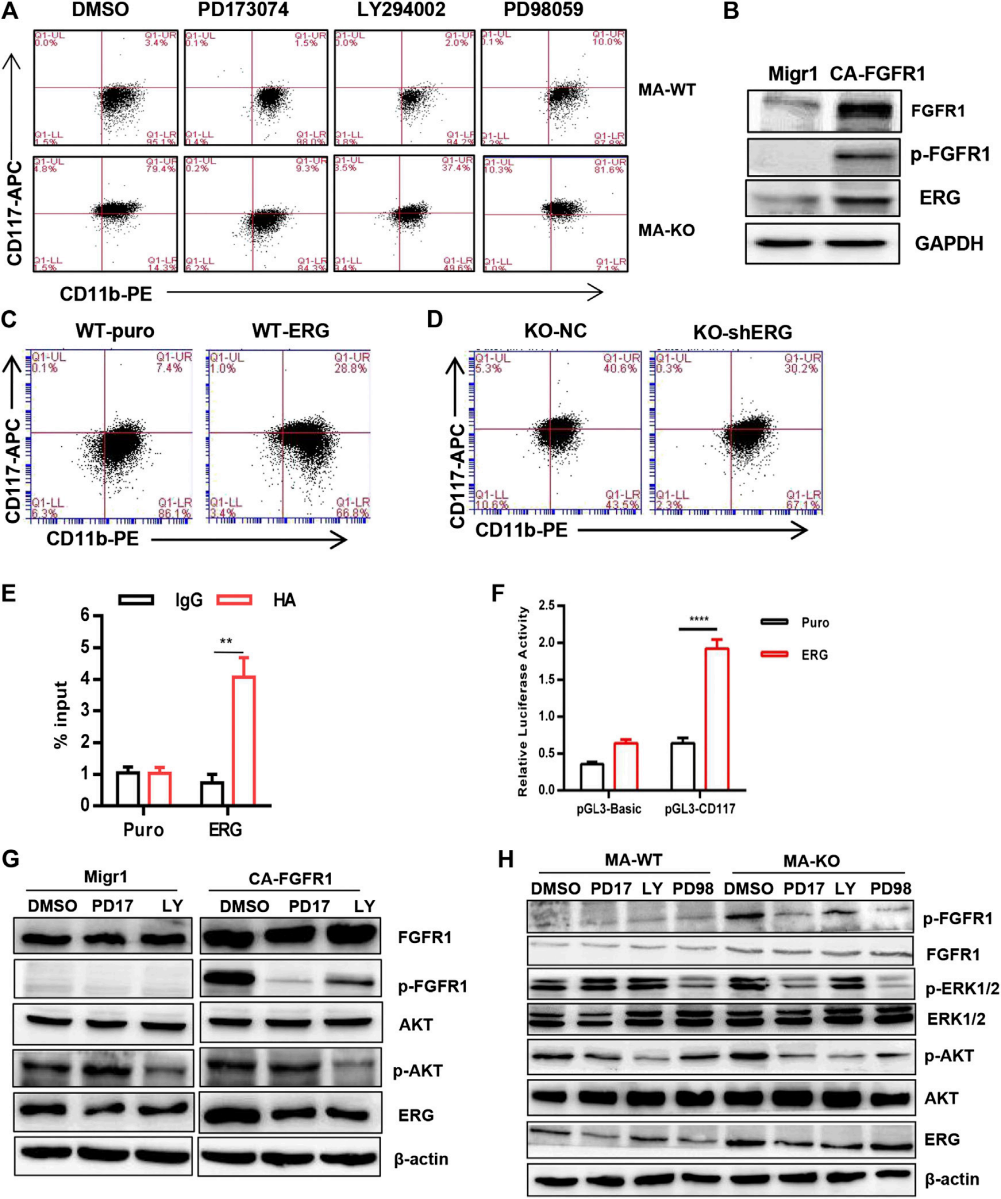
FGFR3 deletion programs CD117^+^ leukemia cells by activating the FGFR1-ERG-CD117 signaling pathway. **(A)** Representative flow cytometric analysis of CD117^+^CD11b^+/low^ in MA-WT and MA-KO cells with FGFR inhibitor (PD173074, 4 μM), AKT inhibitor (LY294002, 50 μM), and MEK inhibitor (PD98059, 50 μM). **(B)** Western blot analysis of ERG expression in 293T cells expressing CA-FGFR1. **(C,D)** Flow cytometric analysis of CD117^+^CD11b^+/low^ in WT-ERG cells **(C)** and KO-shERG cells. **(D,E)** ChIP-qPCR assay of potential binding of ERG at the promoter region of CD117 (n = 4) **(F)** luciferase report assay of transcriptional regulation function of ERG (n = 4). **(G)** Western blot analysis of ERG expression in 293T cells expressing CA-FGFR1 in the presence of signaling inhibitor. **(H)** The expression of *p*-FGFR1, *p*-ERK1/2, *p*-AKT, and ERG in MA-WT and MA-KO cells treated with PD173074 (5 μM), LY294002 (50 μM), and PD98059 (50 μM) for 16 h **p < 0.01, ****p < 0.0001.

To elucidate the molecular mechanism of how FGFR3 deletion promotes the generation of CD117-positive leukemia stem-like cells, RNA-seq analysis and accompanying quantitative reverse transcription-PCR (qRT-PCR) of MA-WT and MA-KO cells were performed. The results showed that some stemness-related genes (such as Hoxa9, Hoxa10, PBX3, etc.) and receptor genes (such as FGFR1 and c-Kit, etc.) were notably upregulated in MA-KO cells compared with MA-WT cells (Figures 3A–E). FGFR1 has been reported in promoting the proliferation of HSCs (de Haan et al., 2003; Magnusson et al., 2005; Zhao et al., 2012) and FGFR1 or its fusion protein drives AML development (Karajannis et al., 2006; Lee et al., 2013; Wang et al., 2016). In order to study whether FGFR1 signaling promotes the generation of CD117-positive leukemia cells, MA-WT and MA-KO cells were treated with FGFR1 inhibitor (PD173074, PD17), AKT inhibitor (LY294002, LY), and MEK inhibitor (PD98059, PD98), respectively. The results showed that PD17 and LY treatment, not PD98 treatment, significantly reduced the percentage of CD117-positive leukemia cells in MA-KO cells compared to MA-WT cells (Figure 4A), suggesting FGFR1 may promote CD117-positive leukemia cells by AKT signaling.

ERG, which is a member of the ETS family, was significantly upregulated in MA-KO cells compared to MA-WT cells (Figures 3A,D). ERG is a key transcriptional factor in the reprogramming of non-blood cells into hematopoietic stem and progenitor cells (Batta et al., 2014; Sugimura et al., 2017). Our results showed that FGFR1 activation upregulated ERG expression in 293T cells (Figure 4B), suggesting that ERG is a downstream target gene of FGFR1 signaling. To further identify whether ERG directly regulates the CD117-positive leukemia stem-like cell pool, we constructed MA-WT-ERG cells which overexpressed ERG in MA-WT cells (Supplementary Figure S3A) and MA-KO-shERG cells which downregulated ERG in MA-KO (Supplementary Figure S3B). The results showed that overexpression of ERG significantly increases the 3-fold percentage of total CD117-positive leukemia cells (Figure 4C). In contrast, knockdown of ERG in MA-KO cells decreases 30 percent of the total CD117-positive leukemia cells (Figure 4D). These findings indicated that the dosage of ERG controls the frequency of CD117-positive leukemia cells.

To further study how ERG promotes the generation of CD117-positive leukemia cells, we analyzed the promoter region of CD117, and found several ERG binding sites (CGGA (A/T). The ChIP-qPCR result from the second pair of primers showed that ERG highly binds to the promoter region of the CD117 gene (Figure 4E). Further, the double luciferase reporter assay proved that ERG transcriptionally upregulates CD117 gene expression (Figure 4F). These results indicated that FGFR3 deletion reprograms leukemia cells into CD117-positive leukemia cells by activating the FGFR1-ERG-CD117 signaling pathway, suggesting that FGFR1 signaling is essential for maintaining the pool of LSCs.

ERG is often regulated by the MAPK/ERK (Huang et al., 2016) or AKT signal (Metzger et al., 2007; Shah et al., 2017). To elucidate how ERG expression was regulated by FGFR1 signaling in MA-KO cells, the inhibitors, including PD17, LY, and PD98, were used to treat FGFR1-active 293T cells and leukemia cells, respectively. The results showed that the inhibition of FGFR1 and AKT, not ERK1/2, significantly reduced FGFR1 activation-induced ERG expression in 293T cells (Figure 4G) and leukemia cells (Figure 4H), suggesting that FGFR1 upregulates ERG expression by activating PI3K/AKT signaling.

### ERG Dosage Is Essential for the Pathogenicity of Leukemia Cells

**FIGURE 5:**
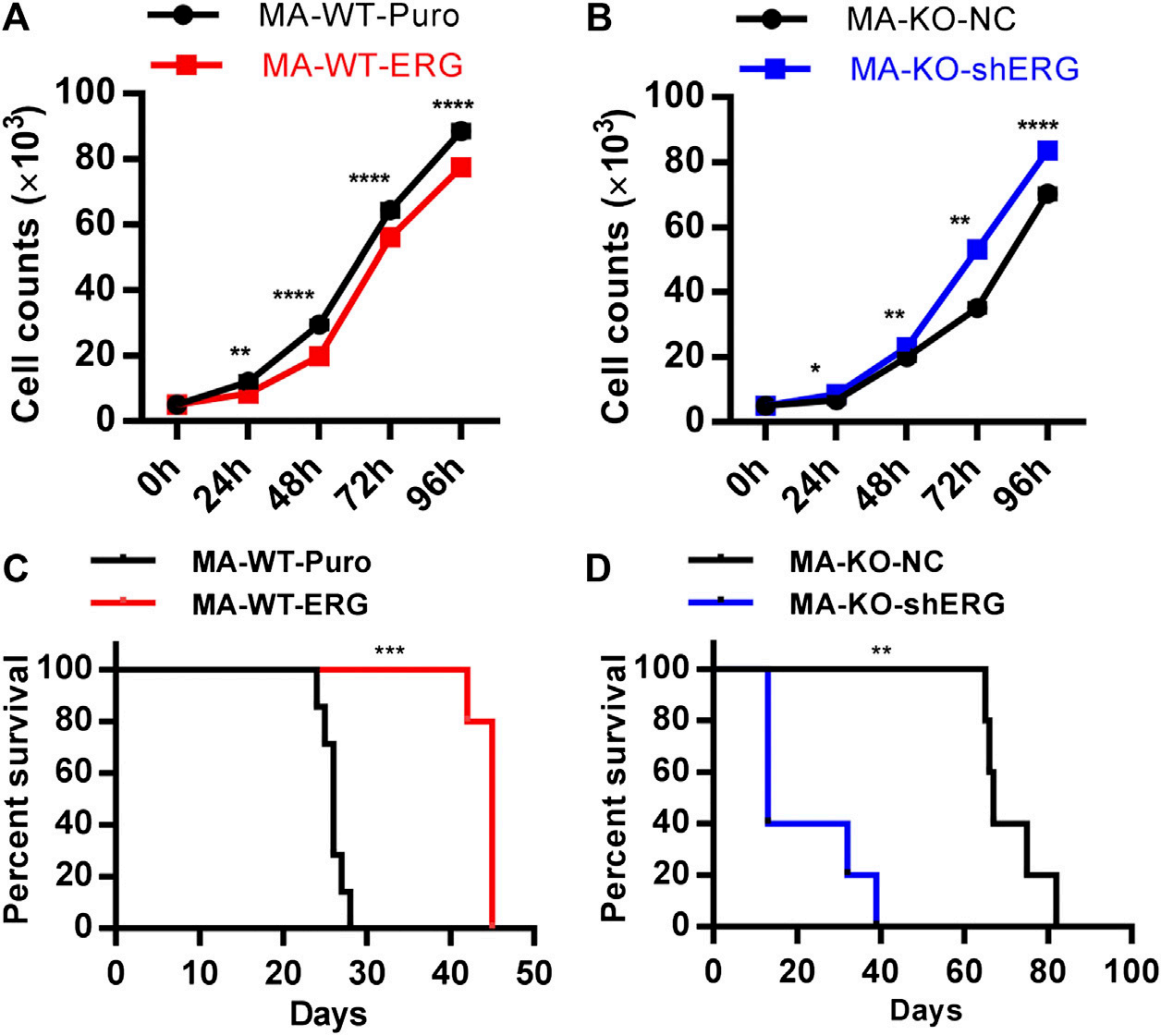
ERG dosage is essential for the pathogenicity of leukemia cells. **(A)** The growth curve of MA-WT-ERG cells compared to the corresponding control cells. **(B)** The growth curve of MA-KO-shERG cells compared to the corresponding control cells. **(C)** Kaplan-Meier curves are shown for leukemogenesis in two groups of mice transplanted with MA-WT-puro cells (n = 7), and MA-WT-ERG cells (n = 5). **(D)** Kaplan-Meier curves are shown for leukemogenesis in two groups of mice transplanted with MA-KO-NC cells (n = 5), and MA-KO-shERG cells (n = 5). *p < 0.05, **p < 0.01, ***p < 0.001, ****p < 0.0001.

The previous study showed that ERG reprograms leukemia cells into CD117-positive leukemia stem-like cells. The further studies indicated that overexpression of ERG in MA-WT cells inhibited cell growth at the indicated time (Figure 5A). In contrast, ERG knockdown in MA-KO-shERG cells promoted cell growth at the indicated time (Figure 5B). *In vivo* transplantation further confirmed that the survival time was significantly extended in MA-WT-ERG-transplanted mice (Figure 5C) and significantly reduced in MA-KO-shERG-transplanted mice compared with respective control mice (Figure 5D). All the above results indicated that the upregulation of ERG increases the frequency of CD117-positive leukemia cells and its dosage is essential for the pathogenesis of MA-driven leukemia cells.

### FGFR3 Deletion Decreased the Expression of the Inflammation Factors and the Extended Survival Time of MA-KO-Transplanted Mice Could Be Neutralized by the Overexpression of CCL3

**FIGURE 6:**
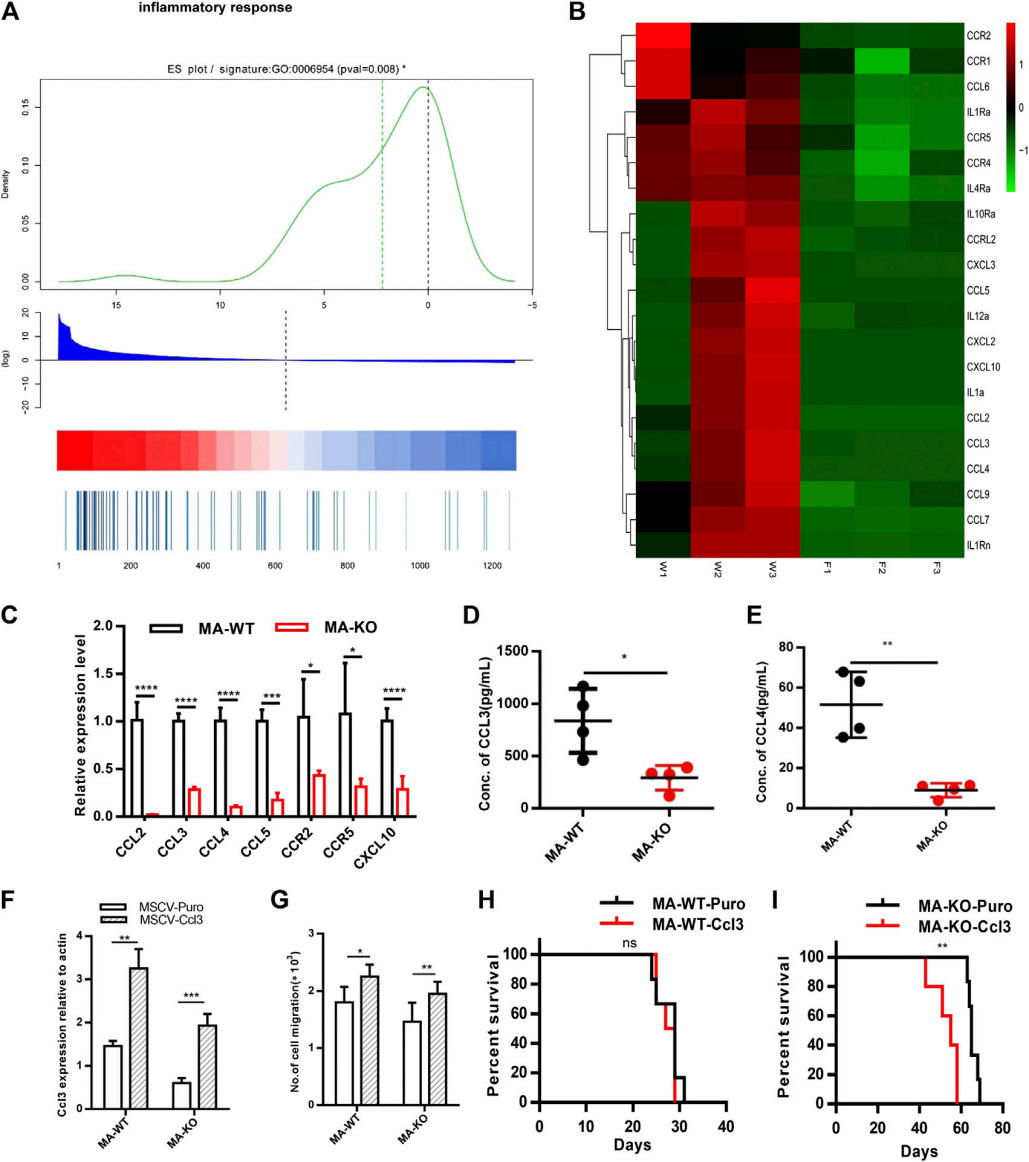
FGFR3 deletion decreases the expression of the inflammation factors and the extended survival time of MA-KO cell-transplanted mice could be neutralized by overexpression of CCL3. **(A)** Gene list rank about inflammatory response genes from RNA-seq data. **(B)** Heat map on the expression level of inflammatory response genes in MA-WT samples (W1, W2, and W3) and MA-KO samples (F1, F2, and F3). **(C)** Differential expression genes from **(B)** by qRT-PCR analysis (n = 4). **(D,E)** The concentration of CCL3 **(D)** and CCL4 **(E)** in BM supernatant of leukemic mice at day 30 after transplantation by ELISA (n = 4). **(F)** Overexpression of CCL3 in MA-WT and MA-KO cells (n = 4). **(G)** Number of cell migration from 100,000MA-WT-CCL3 or MA-KO-CCL3 cells compared with respective control cells (n = 3). **(H,I)** Kaplan-Meier curves are shown for *in vivo* leukemogenesis in two groups of mice transplanted with leukemia cells, including MA-WT-puro cells (n = 6), and MA-WT-CCL3 cells (n = 5) **(H)** as well as MA-KO-puro cells (n = 6), and MA-KO-CCL3 cells (n = 5) **(I)**. *p < 0.05, **p < 0.01, ***p < 0.001, ****p < 0.0001.

**FIGURE 7:**
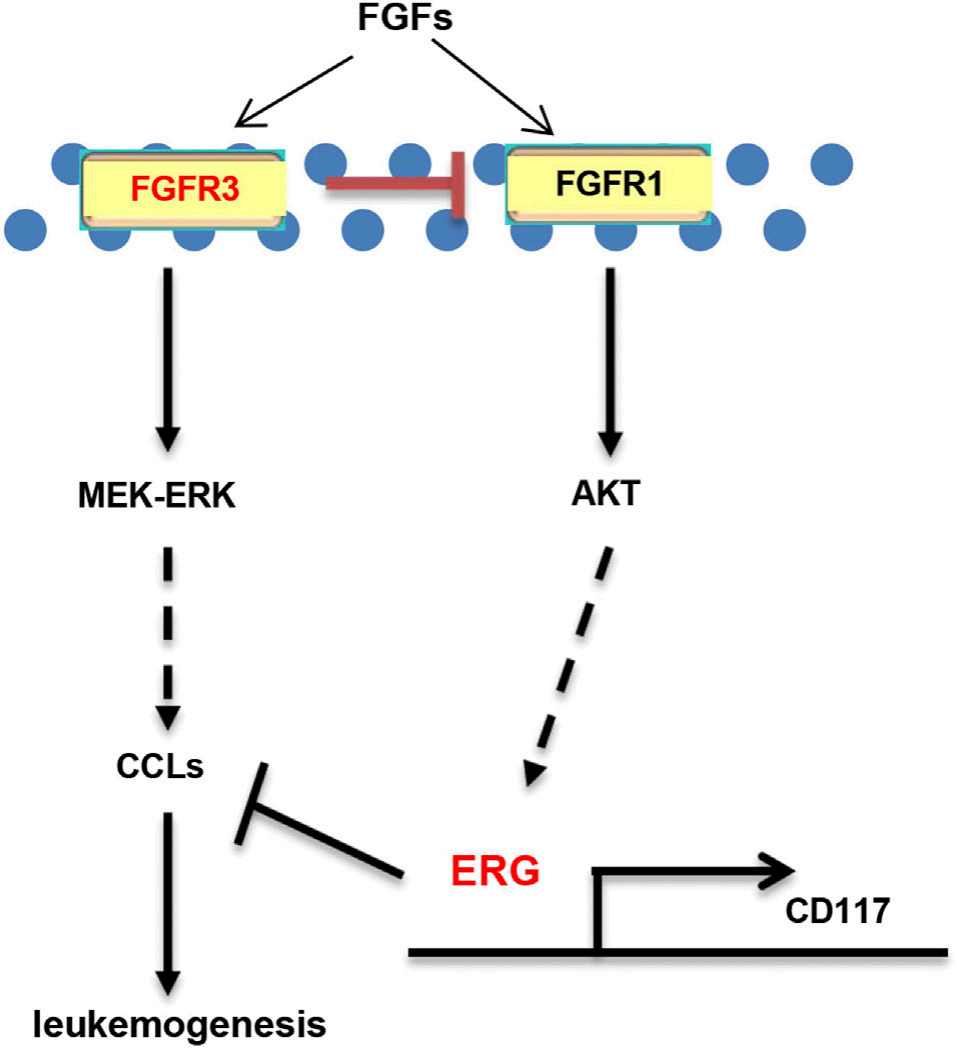
Schematic model of FGFR3 signaling in AML. Schematic model showed that FGFR3 might upregulate CCL expression by activating MEK-ERK1/2 to maintain leukemogenesis. In addition, FGFR3 deletion programs weakly pathogenic CD117^+^ leukemia stem-like cells by activating FGFR1-AKT-ERG-CD117 signaling. Furthermore, ERG does not only promote the stemness of LSCs by upregulating the expression of CD117, but also suppresses *in vivo* leukemogenesis by inhibiting the expression of chemokines genes.

Why are MA-KO cells weakly pathogenic? We found that many enriched inflammatory response genes, such as CCL2, CCL3, CCL4, CCL5, were sharply downregulated in MA-KO cells compared to MA-WT cells (Figures 6A–C). *The in vivo* concentration of CCL3 and CCL4 in the BM supernatant of MA-KO-transplanted mice also significantly decreased compared to that of MA-WT-transplanted mice at day 30 after transplantation (Figures 6D,E), suggesting that FGFR3 signaling promotes the expression of these chemokines genes. This regulatory mechanism was also found in hepatocellular carcinoma cells (Liu et al., 2016) and multiple myeloma (Masih-Khan et al., 2006). CCL3 is an important regulator to maintain CML cells by inhibiting normal hematopoiesis (Baba et al., 2013; Baba et al., 2016), and its expression was regulated by FGFR3 signaling (Masih-Khan et al., 2006). To assess whether CCL downregulation is essential for the weak pathogenesis of MA-KO cells, MA-WT-CCL3 cells and MA-KO-CCL3 cells which overexpressed CCL3 were constructed by retrovirus infection (Figure 6F), and the two cell lines both exhibited a higher migration ability compared to their respective control cells (Figure 6G), suggesting that overexpression of CCL3 performs a better biological function. *In vivo* transplantation demonstrated that the survival time had no notable difference in MA-WT-CCL3-transplanted mice (Figure 6H) and was significantly reduced in MA-KO-CCL3-transplanted mice (Figure 6I) compared with respective control mice, suggesting that the extension of survival time in MA-KO-transplanted leukemic mice is at least partially due to reduced chemokines production. Excitingly, the expression of these chemokines genes (such as CCL2, CCL3, CCL4, and CCL5) was regulated by ERG (Supplementary Figures S4A,B). ERG not only promotes the generation of CD117-positive leukemia cells by transcriptionally upregulating CD117, but is also an important regulator for the weak pathogenesis of CD117-positive leukemia cells by inhibiting the expression of chemokines genes. Collectively, our results indicated that FGFR3 might have dual functions in AML cells. The one is that it upregulates CCL expression by activating MEK-ERK1/2 to maintain leukemogenesis as described previously (Masih-Khan et al., 2006). The other is that it might inhibit the stemness of LSCs by antagonizing FGFR1-AKT-ERG-CD117 signaling (Figure 7).

## Discussion

FGFR3 was identified as a key oncogene in many types of tumors, especially multiple myeloma (Katoh and NaKagama, 2014) and bladder cancer (Wang et al., 2015). FGFR3 overexpression or active mutation promotes tumor cell proliferation, survival, and drug resistance (L’Hote and Knowles, 2005; Iyer and Milowsky, 2013; Javidi-Sharifi et al., 2015). FGFR3 signaling mediated FGF2-induced imatinib resistance in CML (Traer et al., 2014). However, FGFR3 functions in AML as well as AML-LSCs are rarely reported. In our studies, FGFR3-KO mice which were constructed by CRISPR-Cas9 technology display typical bone abnormity as described previously (Deng et al., 1996). Hematopoietic analysis results showed that FGFR3 is dispensable for the functions of HSCs in steady-state hematopoiesis (Ran et al., 2020). To study the functions of FGFR3 in AML, FGFR3-deficient MA-transduced cells were constructed by an infection of retrovirus. Extraordinarily, we found that FGFR3 mediates a novel reprogramming mechanism by which leukemia cells (non-LSCs) could be converted into CD117^+^ leukemia stem-like cells.

In this study, we found that ERG, at the downstream of FGFR1 signaling, is required for FGFR3 deletion-induced reprogramming. ERG highly binds to the promoter region of the CD117 gene and transcriptionally upregulates CD117 gene expression (Figures 4E,F). This is consistent with the previous study which showed that ERG promotes the stem cell signature of leukemia cells (Tsuzuki et al., 2011). Although ERG was always identified as an oncogene in leukemogenesis (Martens, 2011), a study by Mandoli et al. showed that ERG inhibits oncogene expression to protect leukemia cells from apoptosis (Mandoli et al., 2016). Our results indicated that overexpression of ERG in MA-WT cells inhibited cell growth and reduced *in vivo* leukemogenesis. In contrast, downregulation of ERG in MA-KO cells promoted cell growth and enhanced *in vivo* leukemogenesis, suggesting that ERG functions might depend on the expression level of ERG and leukemic type.

Our findings showed that FGFR3 deletion programs leukemia cells (non-LSCs) into CD117^+^ leukemia stem-like cells. It could be a novel regulatory mechanism involving the origin and maintenance of MA-driven LSCs. However, CD117^+^ FGFR3-deficient leukemia cells are not real LSCs because of a defect of FGFR3 downstream signaling and gene expression. Our results indicated that FGFR3 deletion significantly decreased the expression of chemokines and chemokines receptors, suggesting that these genes might be regulated directly by FGFR3 like those in a previous report (Masih-Khan et al., 2006). Further studies showed that the extended survival time of MA-KO cell-transplanted mice could be neutralized by overexpression of CCL3. However, the survival time of MA-WT cell-transplanted mice had no significant difference compared with that of control. One possible reason is that the concentration of CCL3 had attained the threshold because MA-WT cells themselves secret abundant chemokines to bind CCRs on the cell membrane of hematopoietic cells in BM.

In summary, our studies showed that FGFR3 negatively regulates the generation of CD117^+^ leukemia stem-like cells by activating FGFR1-ERG-CD117 signaling. FGFR3-deficient leukemia cells are weakly pathogenic because of downregulation of chemokines genes. Inhibition of FGFR3 will be a promising approach for clinical leukemia therapy.

## Data Availability Statement

The datasets presented in this study can be found in online repositories. The name of the repository and accession numbers can be found below: National Center for Biotechnology Information (NCBI) Sequence Read Archive (SRA), https://www.ncbi.nlm.nih.gov/sra/, SRR13234569, SRR13234570, SRR13234571, SRR13234572, SRR13234573, and SRR13234574.

## Ethics Statement

Animal experiments were approved by the Animal Experiment Ethics Committee of the Basic Medical College of Jilin University.

## Author Contributions

CG, QR, and CS performed experiments and analyzed figures. TZ performed partial experiments. XY, JZ, and SP provided materials and revised the manuscript. YX conceived the project, supervised experiments, analyzed data, and wrote the manuscript. All authors read and approved the final manuscript.

## Funding

This work was supported by National Natural Science Foundation of China (81370640 and 81570149) and funding from Jilin Provincial Science and Technology Department (20180201030YY).

## Conflict of Interest

The authors declare that the research was conducted in the absence of any commercial or financial relationships that could be construed as a potential conflict of interest.
