# Supplementary material for: Loss of FGFR3 Delays Acute Myeloid Leukemogenesis by Programming Weakly Pathogenic CD117-Positive Leukemia Stem-Like Cells
Source: Front Pharmacol. 2021 Jan 29;11:632809. doi: 10.3389/fphar.2020.632809 (PMC7879375; doi:10.3389/fphar.2020.632809)
Supplement: Supplementary file 1 [file datasheet1.docx]

**
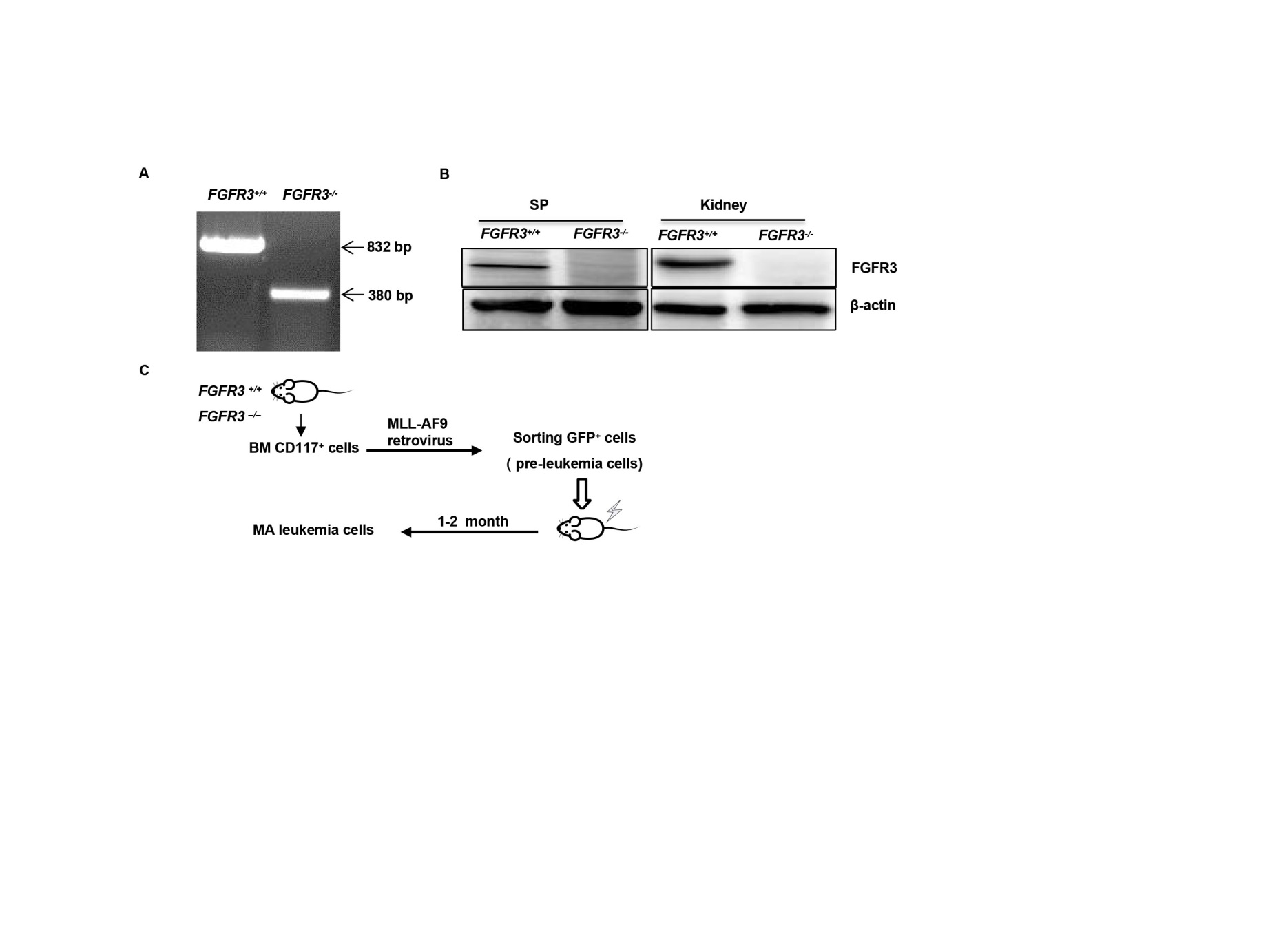
**

**Figure S1. Schematic construction course of FGFR3-deficient MA–driven leukemia cells. (A)** PCR confirmation of FGFR3 from the genomic DNA of WT and KO mouse constructed by Crisp/Cas9 technology. **(B)** WB analysis of FGFR3 deletion in spleen (SP) and kidney from the mice of **(B)**. **(C)** Schematic construction course of FGFR3-deficient MA–driven leukemia cells. Data are representative of at least three independent experiments.


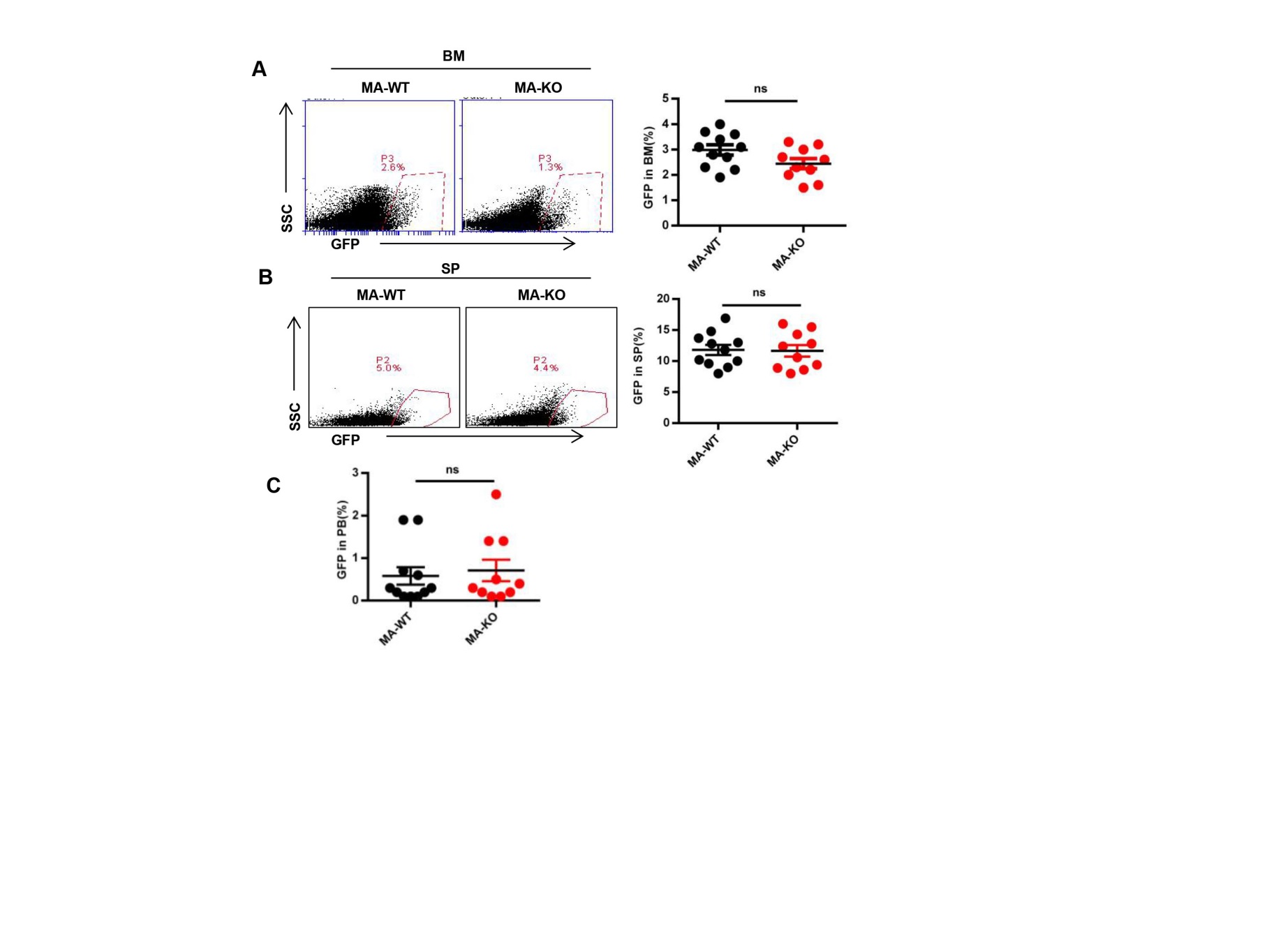


**Figure S2. FGFR3 deletion does not affect homing ability of MA-leukemia cells. (A-C)** Representative dot blots and frequency of GFP^+^ MA-WT and MA-KO cells in BM (A), SP (B) and PB (C) at 24 hours after transplantation.


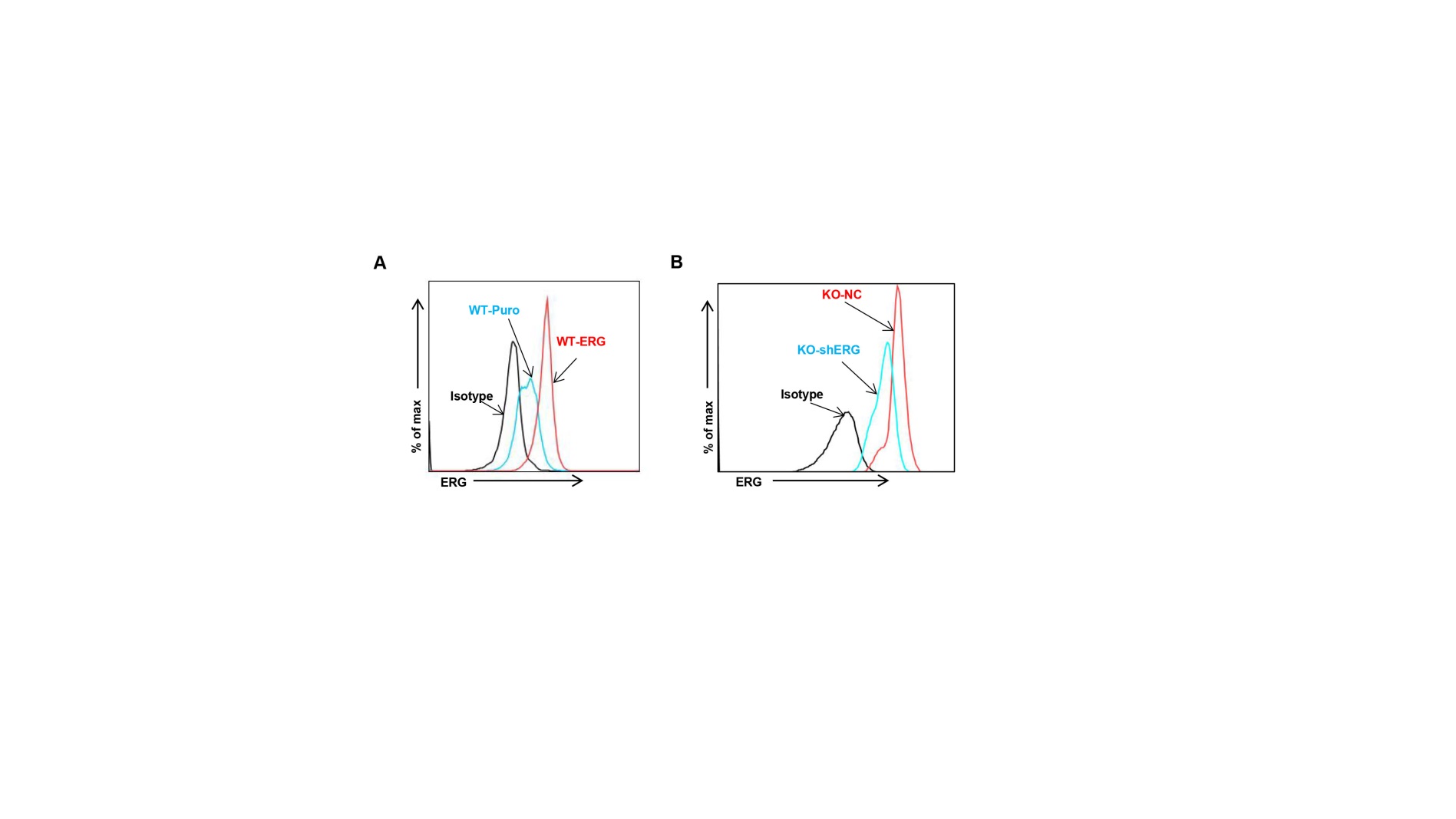


**Figure S3. Construction of MA-WT-ERG cells which overexpressed ERG in MA-WT cells and KO-shERG cells which downregulated ERG in MA-KO cells.**

**(A)** Representative histograms of ERG expression level were shown in MA-WT-ERG cells in which ERG was upregulated by infection of retrovirus containing MSCV-puro-ERG. **(B)** Representative histograms of ERG expression level were shown in KO-shERG cells in which ERG was downregulated by infection of lentivirus containing shERG.


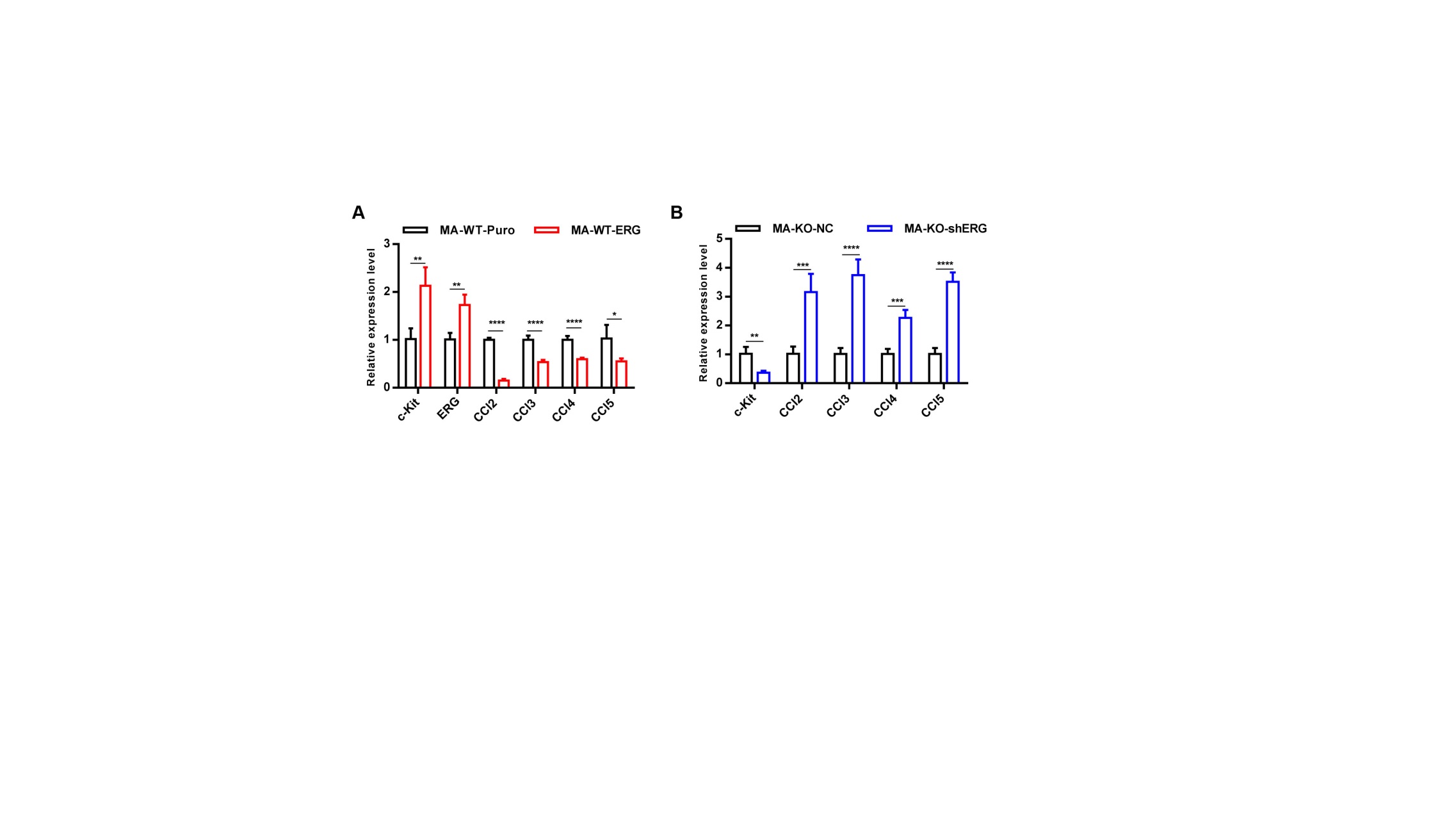


**Figure S4. ERG regulates the expression of inflammation factors. (A-B)** The expression level of ERG, c-Kit, CCL2, CCL3, CCL4 and CCL5 in MA-WT-ERG (**A**) and MA-KO-shERG (**B**) compared to respective control by q-RT-PCR. **P*<0.05, ***P*<0.01, ****P*<0.001, *****P*<0.0001.
